# Do stronger school smoking policies make a difference? Analysis of the health behaviour in school-aged children survey

**DOI:** 10.1093/eurpub/ckw093

**Published:** 2016-06-21

**Authors:** B. Hallingberg, A. Fletcher, S. Murphy, K. Morgan, H.J. Littlecott, C. Roberts, G.F. Moore

**Affiliations:** 1 Centre for the Development and Evaluation of Complex Interventions for Public Health Improvement, School of Social Sciences, Cardiff University, Cardiff, United Kingdom; 2 Social Research and Information Division, Cathays Park, Cardiff, United Kingdom

## Abstract

**Background**: Associations of the strength of school smoking policies with cigarette, e-cigarette and cannabis use in Wales were examined. **Methods:** Nationally representative cross-sectional survey of pupils aged 11–16 years (*N*=7376) in Wales. Senior management team members from 67 schools completed questionnaires about school smoking policies, substance use education and tobacco cessation initiatives. Multi-level, logistic regression analyses investigated self-reported cigarette, e-cigarette and cannabis use, for all students and those aged 15–16 years. **Results:** Prevalence of current smoking, e-cigarette use and cannabis use in the past month were 5.3%, 11.5% and 2.9%, respectively. Of schools that provided details about smoking policies (66/67), 39.4% were strong (written policy applied to everyone in all locations), 43.9% were moderate (written policy not applied to everyone in all locations) and 16.7% had no written policy. There was no evidence of an association of school smoking policies with pupils’ tobacco or e-cigarette use. However, students from schools with a moderate policy [OR = 0.47; 95% (confidence interval) CI: 0.26–0.84] were less likely to have used cannabis in the past month compared to schools with no written policy. This trend was stronger for students aged 15–16 years (moderate policy: OR = 0.42; 95% CI: 0.22–0.80; strong policy: OR = 0.45; 95% CI: 0.23–0.87). **Conclusions:** School smoking policies may exert less influence on young people’s smoking behaviours than they did during times of higher adolescent smoking prevalence. Longitudinal studies are needed to examine the potential influence of school smoking policies on cannabis use and mechanisms explaining this association.

## Introduction

Tobacco use is commonly initiated during youth.^1^ Hence, recent decades have seen growing emphasis on preventing uptake of smoking among young people.^2^ Interventions to influence adolescent smoking are often delivered via schools because they provide opportunities to reach most young people, while the norms and environments of schools’ can influence risk behaviours.^3^^,^^4^ The ‘Health Promoting Schools’ framework, endorsed by the World Health Organization,^5^ consistent with Ottawa Charter principles emphasizing the need to go beyond simplistic health education and toward creating healthier environments,^6^ advocates multi-level approaches to health improvement, focused on integration of health into the curriculum alongside changes to the school’s social and physical environment. Environmental change interventions have demonstrated significant positive effects on a range of outcomes, including tobacco use.^7^

One key strategy for changing school social environments is through written policies. These can play an important role in establishing and communicating a school’s ethos, in terms of norms for acceptable and unacceptable ways for staff, students and others to behave within the school environment.^8^ Changing school policy has been described as low cost, realistic and easy to address^9^ and many schools have adopted formal written smoking policies.^10^ Earlier studies investigating school substance use policies have shown that universal smoking bans and restrictions are associated with a lower likelihood of smoking behaviour and smoking prevalence among youth.^11^ For example, Moore *et al.*^9^ found that having a written smoking policy for all students, teachers and other adults on school premises was associated with lower likelihood of daily and weekly smoking.

However, weaker associations between school smoking policies and tobacco use have been observed in more recent studies.^11^ In part this may be because school smoking policies have become more common and more consistent in their universality, perhaps limiting variance in practice between schools.^9^^,^^12^^,^^13^ However, national policies to ‘denormalize’ smoking, and limit its visibility to children, such as smoke-free legislation, may mean that schools operate within a macro-system in which smoking is already heavily denormalized,^14^ while adolescent smoking rates are now at an all-time low.^15^^,^^16^ Given the growing denormalization of smoking in front of children,^17^ adults may now be less likely to use tobacco on or near school grounds than during earlier studies. Furthermore, young people who continue to smoke in contemporary society do so despite it being widely stigmatized within society and hence may be less influenced by norms within the school environment. As such, the capacity for strong policies to achieve further gains in reducing youth smoking may have diminished over time.

However, no previous studies have looked beyond effects on smoking and toward understanding secondary effects on other substances. Smoking clusters with other risky behaviours^18^ and is often considered a ‘gateway’ into future use of illicit substances such as cannabis.^19^^,^^20^ However, while smoking tobacco is increasingly denormalized, strong government policies on tobacco have been accompanied by mixed messages on cannabis, and it is unclear whether cannabis use has declined at the same rate as tobacco use. Internationally, legislation surrounding cannabis use has become more rather than less permissive, including legalization in some jurisdictions. Perhaps arising from these mixed messages, there is some evidence that young people view cannabis as less harmful than tobacco or even e-cigarettes.^21^ Hence it is plausible that the maintenance of non-smoking norms and norms against substance use more broadly by schools may have effects on cannabis use, which has not been subjected to the same level of policy denormalization.

Emerging international evidence also indicates increasing numbers of young people who have never used tobacco are experimenting with electronic (e)-cigarettes.^22–24^ Some have expressed concern regarding the visibility of e-cigarettes in places where the use of tobacco has been banned.^25^ According to advocates of the renormalization hypothesis,^26^ the appearance of smoke-like vapour in public places will ultimately lead to a regrowth in smoking rates through increasing the extent to which smoking is seen as a normative behaviour. However, it may also be that contexts with more permissive approaches to tobacco use give rise to more widespread experimentation with e-cigarettes, as a safer means of experimenting with nicotine use.

The objective of this study is therefore to identify the extent to which students’ cigarette, e-cigarette and cannabis use are associated with school smoking policies. This study conducts a multilevel analysis, combining school-level data on smoking policies with student-level data on substance use from a nationally representative cross section of young people aged 11–16 years in Wales.

## Methods

### Sample

Data were derived from the 2013/14 Welsh Health Behaviour in School-aged Children (HBSC) survey, a cross-sectional study of Welsh secondary school students aged 11–16 years in a nationally representative sample of secondary schools. The Welsh survey is part of an international survey representing 44 countries supported by the World Health Organization to monitor national and international changes in health behaviours among school-age students every four years.^27^^,^^28^ A two-stage sampling procedure was used. First, maintained and independent secondary schools in Wales were stratified by local authority and eligibility for free school meals (FSMs). Schools were selected using probability proportionate to size, with the sample disproportionately stratified to allow analysis at local health board level. An invitation letter was sent to school head teachers to take part in the HBSC survey and was followed up with telephone calls. Second, participating schools were asked to randomly select one class (∼25 students) from each school year 7–11 (i.e. 11–16 years old). Data were collected between November 2013 and March 2014 in the classroom under examination conditions. Teachers were present during data collection but remained at the front of the classroom so they could not see students’ responses. In 67 schools, members of the senior management team also completed a school environment questionnaire on the content of school health policies. Schools received £150 to cover any costs incurred due to participating.

### Measures

#### Socio-demographics

Students reported their sex, year and month of birth and ethnicity [White; Mixed Race; Asian or Asian British; Black or Black British; Chinese or Other (categorized as white/BME)]. The Family Affluence Scale (FAS) was used to indicate family-level socioeconomic status.^29^ The measure is the sum of six survey items which asked students whether they have their own bedroom, how many family holidays they took in the past year, if their family owns a dishwasher, how many bathrooms are in their home and how many computers and cars their family own. Items on dishwashers and bathrooms were introduced in 2013, due to concerns that some items (i.e. computer ownership) became less differentiated by socio-economic status over time. At the school level, the modified six-item measure correlated slightly more strongly to the percentage of students eligible for FSM entitlement (*r* = −0.84, *P* <0.001), than the original four-item FAS measure (*r* = −0.76, *P* < 0.001), and hence the six-item measure was used.

#### Substance use measures

##### Cigarette use

Smoking behaviour was assessed using the question ‘How often do you smoke at present?’ (responses: ‘every day’; ‘at least once a week but not everyday’; ‘less than once a week’ and ‘I do not smoke’). For analyses, a binary variable was created that compared those stating ‘I do not smoke’ against all others.

##### E-cigarette use

E-cigarette use was based on the question ‘Have you ever used or tried electronic cigarettes?’ (responses: ‘I have never used or tried e-cigarettes’; ‘I have used e-cigarettes on a few occasions (1–5 times)’ and ‘I regularly use e-cigarettes (at least once a month)’. A binary variable was created that compared ever e-cigarette users to never users.

##### Cannabis use

Cannabis use was measured by asking students to report the number of days they used cannabis in the past 30 days (responses: ‘never’; ‘1–2 days’; ‘3–5 days’; ‘6–9 days’; ‘10–19 days’; ‘20–29 days’ or ‘30 days (or more)’. A binary variable was created to indicate those who used any cannabis in the past 30 days.

#### School environment measures

##### School smoking policies

Schools reported whether they had a written policy in place for smoking and tobacco use and whether it prohibited tobacco use on: school grounds during school hours; school grounds outside school hours; in private vehicles on school grounds and/or, school events off school grounds. They were also asked whether their policy applied to everyone, including students, staff, families and/or visitors. A three-level categorical variable was derived to indicate the strength of school smoking policies. These were categorized as ‘no written policy’, ‘moderate’ (a written policy but not applied to everyone in all locations) and ‘strong’ (a written policy that applied to everyone in all locations).

##### Substance use education and tobacco cessation initiatives

In all models other school-level approaches aimed at reducing students’ substance use, such as tobacco cessation initiatives and substance use education, are controlled for as school smoking policies may reflect wider school norms around substance use. Schools were asked if they provided any tobacco, alcohol or drug education to students in years 7, 8, 9, 10 and 11. A scale was created by adding the number of year groups that received education on the various substances and divided by three to indicate the average number of years within a school that received substance use education. A binary variable indicated whether schools provided tobacco cessation initiatives.

##### FSM entitlement

The Welsh Government provided information on the percentage of students entitled to FSMs within schools. This was used to generate a continuous variable to indicate school-level socioeconomic status. A higher percentage represented more deprived schools.

### Statistical analyses

Multi-level, binary logistic regression models (students nested within schools) were used to investigate cigarette use, e-cigarette use and cannabis use. Three models were used for each substance use outcome and applied to both the entire student sample and a sub-sample of students aged 15–16 years with complete data. For each model, a null model was first developed and included schools as a random effect. For the second model, individual-level variables (age, gender, ethnicity and FAS) were then entered as fixed effects. Finally, school-level predictors (school smoking policy strength, substance use education, tobacco cessation initiatives and FSM entitlement) were entered into the model. All analyses were conducted in STATA (v.14.0). Analyses are conducted with the whole sample (i.e. all year groups) and with young people aged 15–16 years; the latter to enhance comparability with an aforementioned study of the link between school policy and tobacco use which included only 15–16-year olds.^9^ Data for the one school which did not provide full details of their school smoking policy were excluded from regression analyses.

## Results

Questionnaires were completed by 7376 students (49.1% girls and 50.9% boys) at the 67 schools. Table 1 provides a description for individual level variables for the entire student sample and for students aged 15–16 years. Overall, 5.3% of students were identified as current cigarette smokers, with a small percentage of students reporting smoking daily (2.3%) or weekly (0.9%). The prevalence of e-cigarette ‘ever use’ was 11.5%, while 2.9% of students reported that they had used cannabis in the past month. For all substance use outcomes, prevalence rates among students aged 15–16 years were around double the rate of the sample as a whole.

**Table 1:** Description of sociodemographics and substance use outcomes for the entire sample and those aged 15–16 years

Characteristics	Entire sample	15–16 years only
Age, years,^a^ M (SD)	13.7 (1.4)	15.6 (0.4)
FAS,^b^ M (SD)	15.0 (2.3)	15.0 (2.3)
Male/female, % (*n*)	50.9 (3743)/49.1 (3607)	50.9 (937)/49.1 (903)
White/BME, % (*n*)	92.7 (6808)/7.3 (534)	93.7 (1729)/6.3 (116)
Smoking status		
Smoke daily	2.3 (169)	5.1 (93)
Smoke weekly	0.9 (68)	2 (37)
Smoke <1/week	2.1 (152)	3.7 (68)
Don’t smoke	94.7 (6969)	89.2 (1644)
Current smoker (yes/no)	5.3 (389)/94.7 (6969)	10.8 (198)/89.2 (1644)
E-cigarette use		
A few times (1–5)	10.1 (743)	17.5 (320)
At least once a month	1.3 (97)	2.3 (42)
Any e-cigarette use (yes/no)	11.5 (840)/88.5 (6451)	19.8 (362)/80.2 (1465)
Cannabis use past month (yes/no)	2.9 (204)/97.1 (6850)	7.1 (125)/92.9 (1644)

a *n* = 7345.

b *n* = 7200.

Of the 67 schools, 37 (55.2%) provided tobacco cessation initiatives, 41 (65%) delivered education on alcohol, tobacco and drug use to all students in years 7–11 and 56 (83.6%) had a written school smoking and tobacco policy. An average of 15.9% (SD = 8.7) of students within schools were entitled to FSM. All but one school provided all the information needed to generate an indicator of school smoking policy strength. Of the remaining 66 schools, 26 (39.4%) had a strong policy, 29 (43.9%) had a moderate policy and 11 (16.7%) had no written policy.

As indicated in table 2 for the entire sample, individual and school-level characteristics were associated with some substance use behaviours. Older age was associated with a greater likelihood of all substance use outcomes. Females were more likely to be current smokers and to have used cannabis in the past month. At the school level, having a greater percentage of students entitled to FSM was associated with a greater likelihood of e-cigarette use.

**Table 2:** Odds ratios and 95% confidence intervals from logistic regression analyses of cigarette use, e-cigarette use and cannabis use, for the entire sample and 15–16-year-old students

		Entire sample			Students 15–16 years		
		Cigarette use	E-cigarette use	Cannabis use	Cigarette use	E-cigarette use	Cannabis use
		(*N* = 6538)	(*N* = 6484)	(*N* = 6266)	(*N* = 1629)	(*N* = 1620)	(*N* = 1568)
Individual level variables	Age	**1.8 (1.65–1.97)**	**1.64 (1.54–1.74)**	**2.47 (2.13–2.87)**	**1.69 (1.15–2.50)**	**1.73 (1.25–2.39)**	**1.96 (1.20–3.18)**
	Female	**1.39 (1.11–1.74)**	0.90 (0.76–1.07)	**1.44 (1.05–1.98)**	**1.78 (1.28–2.47)**	1.08 (0.83–1.40)	**1.57 (1.04–2.35)**
	Ethnicity	0.80 (0.47–1.34)	0.98 (0.70–1.38)	1.29 (0.69– 2.41)	0.79 (0.37–1.69)	0.90 (0.50–1.62)	1.09 (0.47–2.53)
	FAS	0.96 (0.91–1.01)	0.99 (0.96–1.03)	0.95 (0.89–1.02)	0.94 (0.88–1.01)	1.00 (0.94–1.06)	0.97 (0.89–1.06)
School level variables	FSM	1.07 (0.93–1.24)	**1.40 (1.18–1.65)**	1.22 (0.98–1.52)	0.98 (0.81–1.18)	**1.25 (1.02–1.54)**	1.05 (0.82–1.35)
	Tobacco Cessation	1.09 (0.81–1.49)	0.72 (0.51–1.02)	0.79 (0.50–1.24)	0.94 (0.64–1.40)	0.76 (0.49–1.17)	0.76 (0.46–1.23)
	Education	0.98 (0.85–1.14)	1.04 (0.87–1.25)	1.18 (0.93–1.51)	0.96 (0.79–1.17)	1.05 (0.84–1.31)	**1.40 (1.03–1.89)**
	Policy -Weak						
	Moderate	0.83 (0.55–1.25)	0.64 (0.39–1.03)	**0.47 (0.26–0.84)**	0.83 (0.49–1.42)	0.66 (0.37–1.18)	**0.42 (0.22–0.80)**
	Strong	0.87 (0.57–1.33)	0.82 (0.50–1.34)	0.61 (0.33–1.11)	0.93 (0.54–1.62)	0.77 (0.42–1.41)	**0.45 (0.23–0.87)**
ICC: constant only		0.04	0.12	0.05	0.04	0.11	0.11
ICC: level 1 variables		0.05	0.14	0.05	0.04	0.12	0.12
ICC: level 1 and 2 variables		0.03	0.09	0.05	0.03	0.09	0.6

For the whole sample, percentages reporting smoking were 5.5% (*N* = 64), 5.2% (*N* = 167) and 5.6% (*N* = 158), respectively, in schools with no policy, moderate policy or strong policy. For cannabis use, percentages were 4.1% (*N* = 46), 2.5% (*N* = 77) and 2.9% (*N* = 78), and for ever e-cigarette use 12.6% (*N* = 146), 11.9% (*N* = 379) and 10.9% (*N* = 307). For over 15s, percentages reporting smoking were 11.5% (*N* = 30), 9.9% (*N* = 81) and 12.0% (*N* = 87) in schools with no policy, moderate policy or strong policy. For cannabis use, percentages were 12.6% (*N* = 31), 6.0% (*N* = 47) and 6.5% (*N* = 46), respectively, and for ever e-cigarette use 23.8% (*N* = 62), 20.3% (*N* = 164) and 18.5% (*N* = 133). Students who attended schools with a moderate smoking policy were significantly less likely than students from schools with no written smoking policy to use cannabis in the past month, though there was no association with tobacco or e-cigarette use. As indicated by the ICCs, school-level clustering was substantially greater for e-cigarettes and cannabis than for tobacco use in models excluding school-level variables, particularly for older adolescents. Among 15–16-year olds, inclusion of school level variables substantially reduces the ICC for cannabis, suggesting that half of school-level variance is explained by the included variables.

Among students aged 15–16 years, females were more likely to be current smokers and use cannabis in the past month, while higher FSM entitlement was associated with a greater likelihood of e-cigarette use. Schools with a moderate or strong smoking policy had lower rates of cannabis use compared to schools with no written smoking policy, though as for the full sample, there was no association with tobacco or e-cigarette use. The provision of tobacco, alcohol and drug use education across a greater number of school years was associated with an increased likelihood of cannabis use.

## Discussion

Compared to earlier studies,^9^ this study indicated an increased uptake of smoking policies within schools, in line with growing societal anti-smoking norms. In the 1998 HBSC survey, only 16.4% of schools had a strong smoking policy,^9^ while in this study, 39.4% schools had a strong smoking policy. Smoking prevalence among students in Wales has also dramatically decreased. In this study, 2.3% of the entire sample and 5.1% of students aged 15–16 years smoked daily compared to approximately one in five 15–16-year olds in 1998. While a key function of school smoking policies has been to communicate a strong non-smoking norm,^30^ schools now operate within a wider macro-system in which smoking, particularly in spaces where children are present,^17^ is increasingly denormalized. Policies banning smoking on or near school grounds may therefore make less difference to the visibility of smoking in these spaces than they once did. Furthermore, young people who continue to take up smoking despite the widespread stigma associated with smoking in contemporary UK society are perhaps also less influenced by school norms.

To our knowledge, this study is the first to examine the relationship between school smoking policies and other substance use outcomes, such as cannabis and e-cigarette use. Although school smoking policies were not associated with student’s e-cigarette use, a stronger school smoking policy was associated with less cannabis use, particularly among those aged 15–16 years. The mechanisms through which school smoking policies might influence cannabis use are unclear. However, this may arise in part from the greater ambiguity in public health messages around cannabis use compared to those surrounding tobacco use. In the UK, cannabis was reclassified as a Class C (the category of drug associated with the lowest penalties for sale and use, due to lower perceptions of harm), before being returned to Class B (an intermediate category between Class A drugs such as heroin, which are associated with the strongest penalties, and Class C drugs such as ketamine and anabolic steroids) status in 2008; a decision which was again debated in 2012, with a Home Affairs Committee tied on a vote regarding reclassification to Class C.^31^ In the USA, a number of states have recently legalized cannabis is widely perceived by young adults to be significantly less harmful, and more socially acceptable, than tobacco.^21^

Notably, in schools with more comprehensive coverage of substance use education, cannabis use was more likely among 15–16-year olds (though not for the whole sample). However, this likely reflects reverse causality; schools with greater perceived problems with cannabis use may be more likely to increase health education coverage as a response to this. Experimentation with e-cigarettes for the whole sample, and cannabis use for older adolescents, were highly clustered within schools, perhaps indicating a higher degree of influence by aspects of the school environment such as peer relationships than for tobacco. Associations between tobacco use and e-cigarette use remains highly ambiguous. Experimentation with e-cigarettes has increased among adolescents, although this has not been accompanied by widespread regular use, and e-cigarettes do not appear to be making a major contribution to young people’s nicotine addiction.^17^

While the large, nationally representative sample is a strength, data for this study are based on self-reports of substance use. As with any cross-sectional study, the current results should be interpreted with caution. Reverse causality cannot be ruled out: for example, schools with less prevalent substance use may face less resistance from students, teachers and other adults in implementing strong non-smoking policies. Future research should examine the impact of school smoking policy change on student’s substance use over time to aid interpretation of any directional relationships. While this study did examine the strength of school smoking policy it did not investigate the enforcement of policy and consequences from breaking school policy which have been associated with lower student smoking rates in other studies.^9^^,^^32^^,^^33^ It is possible that details of the content of these policies, which were not captured in this study, may have moderated their effects. Finally, associations from the multi-level analyses may be caused from unmeasured differences between schools and student characteristics confounded with smoking policy. The power to detect impacts of school-level variables on young people’s smoking has diminished over time as smoking has become confined to a small minority of students and retesting these associations with larger samples is perhaps important.

Nevertheless, the study has important implications for policy and practice. Firstly, school smoking policies appear to make a less important contribution to the reduction of smoking uptake than they did when smoking was highly prevalent among young people. It is perhaps the case that within a macro-context in which smoking is already heavily denormalized, the potential contribution of school policies to further reducing smoking has been reduced. Hence, revisiting our assumptions about how best to influence young people’s smoking uptake within this changed macro-context is important. Nevertheless, despite the apparently declining effect of smoking policies on tobacco use itself, schools should be advised to continue to implement such policies, due to their potential effects on other substances such as cannabis. In some countries, including Wales, policy debates are moving towards extending smoke-free legislation to some outdoor spaces, such as on or near schools or on children’s playgrounds. Hence, all schools may in time become entirely smoke-free spaces, regardless of internal school policies. The failure of the Public Health Wales Bill to pass through the Welsh Assembly due in large part to the retention of controversial legislation on e-cigarettes within this has delayed such a move in Wales.^34^ Nevertheless, examining the effects of further strengthening of smoke-free public place policies on youth tobacco use, and on cannabis use, is important. Given that cannabis use appears to be more sensitive than tobacco use to norms within the school gates, wider societal efforts to address ambiguities regarding the harms of cannabis are perhaps also needed. Relationships of e-cigarettes with tobacco use remain ambiguous and contested, and further research is needed to understand the substantial clustering of e-cigarette within schools, and the nature of their relationships with other substances.

Key points

- School smoking policies play a crucial role in setting behavioural norms and guiding student behaviour.
- National policies have aimed to ‘denormalize’ smoking within society as a whole and an increasing number of schools have implemented school smoking policies in the last two decades; however, this has also been accompanied by mixed messages on cannabis and e-cigarette use.
- More recent studies that have investigated the impact of school smoking policies on student smoking show weaker associations compared to earlier studies that took place when tobacco use was more prevalent among students.
- While strong school smoking policies were not associated with student’s smoking or e-cigarette use in this study, they were associated with less cannabis use, particularly among students aged 15–16 years.
- Schools should continue to implement strong school smoking policies due to their potential effects on other substances. There is a need to better understand how to influence the minority of young people who still take up smoking in contemporary society.
